# Recent advances in electroconvulsive therapy in clinical practice and research

**DOI:** 10.12703/r/12-13

**Published:** 2023-06-07

**Authors:** Fahad Mukhtar, William Regenold, Sarah H Lisanby

**Affiliations:** 1 Noninvasive Neuromodulation Unit, Experimental Therapeutics & Pathophysiology Branch, National Institute of Mental Health, National Institutes of Health, Bethesda, MD, USA

**Keywords:** Electroconvulsive therapy (ECT), treatment resistant depression (TRD), magnetic seizure therapy (MST), dosing

## Abstract

Electroconvulsive therapy (ECT), the oldest somatic therapy still in use in psychiatry today, remains one of the most effective therapeutic interventions for a wide variety of psychiatric disorders. In this article, we review some of the recent advances in ECT that are currently being researched and implemented in clinical practice. We explore recent studies that point to the potential therapeutic benefit and safety of ECT in COVID-19-related neuropsychiatric complications and special populations (such as the elderly and pregnant persons) that are generally at higher risk of having adverse effects from psychotropic medications. We highlight studies that performed a head-to-head comparison of ECT and ketamine, which has shown promise for treatment-resistant depression and acute suicidality. Researchers continue to explore different ways of using ECT by modifying the treatment parameters to maintain efficacy and decrease side effects. Neurocognitive side effects remain one of the major drawbacks to its use and contribute to the negative stigma of this highly effective treatment. In this regard, we describe attempts to improve the safety of ECT by modifying dosing parameters, novel electrode placements, and the addition of augmenting agents with the aim of decreasing side effects and improving efficacy. This review identifies some of the recent advances in the last few years in ECT research while also highlighting areas where further research is needed.

## Introduction

Since its invention and first use in 1938 by Italian psychiatrists Ugo Cerletti and Lucio Bini^1^, electroconvulsive therapy (ECT) remains one of the most effective interventional tools in psychiatry, outlasting many other psychiatric interventions. Early case reports and observational studies on ECT showed good efficacy in treating severe mental illness, and it quickly made its way into Europe and the United States, replacing chemically induced convulsive therapies that were in use at the time^2^. Due to concerns related to mechanical injuries from motor convulsions, the treatment was refined in the 1950s by the addition of a muscle relaxant and an anesthetic agent to reduce tissue injury^1^. This led to a significant decrease in the prevalence of this complication associated with ECT^3^. 

Despite its efficacy in the rapid treatment of multiple psychiatric disorders and symptoms, ECT remains an unpopular, stigmatized psychiatric treatment, partly due to its cognitive side effects (SEs), including anterograde and retrograde amnesia. Psychiatrists Lancaster, Steinert and Frost introduced the non-dominant hemisphere unilateral electrode placement method that has a superior SE profile compared to the conventional bitemporal (BT) placement^4,5^. Later, the D’Elia placement came to be accepted as the standard right unilateral (RUL) placement^5–7^, yet, cognitive SEs still persist^8^. Importantly, we still have not fully characterized the mechanisms of action and SEs of ECT. Studies have investigated numerous potential therapeutic mechanisms including increased neurogenesis, monoaminergic neurotransmission, activation of the immune system and neuroendocrine activation^9^. 

In this article, we will explore recent advancements in research within the last three years that aim to reduce cognitive SEs of ECT and review studies that attempt to compare ECT with newer treatment modalities. We will also review recent research that explores the following areas: a) potential novel indications for ECT; b) combination of ECT with other treatment modalities; and c) optimization of dosing and administration.

## ECT use in novel indications and special populations

In addition to its common indication in patients with severe treatment-resistant major depression, bipolar disorder, schizophrenia and catatonia, researchers continue to explore the use of ECT in other psychiatric disorders. Researchers have explored the amnestic SE of ECT as a possible therapeutic effect in patients with post-traumatic stress disorder (PTSD) by attempting to disrupt the reconsolidation of traumatic memories^10–12^. Most recently, Tang *et al.* randomized 25 female patients with comorbid depression and PTSD to ECT paired with pre-treatment traumatic memory reactivation and ECT paired with pre-treatment non-traumatic memory reactivation. The authors hypothesized that ECT will decrease traumatic memory after reactivation of the traumatic memory. The results showed no difference in the two treatment groups in both the Modified PTSD Symptom Scale Self Report (MPSS-SR) and the Clinician-Administered PTSD Scale for DSM-5 (CAPS-5) measured immediately post-ECT treatment course and at 3 months follow-up. Importantly, however, both groups had an approximately 35% decrease in MPSS-SR score and a 37% decrease in CAP-5 score at 3 months follow-up. A significant drawback in this study was the inability to assess the moderating effect of improvement in depression scores due to insufficient data^10^.

Some authors have theorized that the improvement in PTSD symptoms after ECT could be due to increased cognitive and adaptive function as a result of increased neuroplasticity and synaptogenesis rather than amnestic SEs^13^. A recent pilot study randomly assigned seven patients diagnosed with depression and comorbid PTSD to receive either low amplitude seizure therapy (LAP-ST), an ECT protocol that has been found to have fewer cognitive SEs, or right unilateral (RUL) ECT^13^. All patients had an improvement in PTSD Checklist (PCL) scores with a mean change in PCL score from 42.5 to 31 in the LAP-ST group and 64.7 to 41 in the RUL group. Future studies are needed to replicate these findings using larger samples and a longer follow-up time to determine long-lasting effects of ECT in patients with PTSD.

Researchers are continuing to explore the safety and use of ECT among the vulnerable elderly population^14^. The Prolonging Remission in Depressed Elderly (PRIDE) study^15^ examined whether a novel continuation algorithm of ECT plus medication (venlafaxine + lithium) was associated with a better relapse rate and SE profile compared with medication only (venlafaxine + lithium) among elderly patients >60 years of age with major depressive disorder (MDD) who achieved remission after an acute course of RUL ultra-brief (RUL-UB) ECT with venlafaxine. The study developed a novel individualized ECT algorithm for the continuation phase comprising once-weekly ECT treatment for 4 weeks and a subsequent treatment (5–24 weeks) schedule that was based on patients’ Hamilton Depression Rating Scale (HDRS) scores. Patients who received continuation ECT + medication had a better HDRS score at the end of the 24-week follow-up compared to the medication-only group^15^. In addition, most of the neurocognitive SEs, such as deficits in short and long-term memory, cognitive flexibility, phonemic fluency and recognition of learned words, that developed in both treatment groups were short-lived with most patients returning to baseline within 1–6 months and there was no statistically significant difference on the mini-mental state examination^16^. In a secondary analysis, the authors also found RUL-UB ECT to be very effective with a 75% remission rate in elderly patients with moderately severe MDD, a sub-population that has not been investigated extensively for ECT response^17^.

ECT has been investigated in the management of Parkinson’s disease with psychiatric manifestation and/or comorbid psychiatric conditions, especially since anti-psychotics and anti-parkinsonian medications could worsen parkinsonian symptoms and psychotic symptoms respectively. A study of 12 patients with Parkinson’s disease and comorbid psychiatric illnesses including depression, hallucinations, delusions and impulse control disorder^18^ found an improvement in both psychiatric and motor symptoms of Parkinson’s disease post-ECT. Similarly, another study of 15 patients with Parkinson’s disease with psychiatric symptoms reported improvement in mentation, behavior, mood, performance of daily activities, motor status and psychiatric symptoms at the end of 12 sessions of ECT and at 1-month follow-up^19^.

ECT has long been a treatment option during pregnancy, especially when there is no safer treatment; however, its use remains limited, partly due to the lack of studies that establish safety and provide accurate guidelines that would ensure fetal well-being^20^. Evidence from case reports and case series have reported changes in fetal heart rate (FHR) activity associated with ECT and guidelines have generally advised auscultation of FHR for pregnancies <24 weeks and non-stress tests (continuous FHR) for pregnancies >24 weeks, pre- and post-ECT, respectively^20,21^. A recent study performed continuous FHR during and for 10 minutes following ECT treatment in five women with severe mood disorder during their course of ECT treatment^22^. A total of 34 fetal monitoring recordings were obtained in 44 ECT sessions and only four were associated with FHR decelerations (decreased heart rate > 30 bpm) with none of the recordings having a nadir below 90 bpm. The decelerations resolved spontaneously within 4 minutes and the study did not have any negative fetal outcomes. This study, though small in size, provides supporting evidence for the safety of ECT, particularly in pregnant women with very severe mood and psychotic illnesses that may not be amenable to pharmacotherapy. In such cases, ECT may be a safe treatment alternative.

Although there were reductions in ECT utilization during the COVID-19 pandemic, it was found useful as a treatment modality in a few cases of new-onset COVID-19-related neuropsychiatric complications. A 51-year-old woman who developed her first episode of depression, psychosis and catatonia following COVID-19 infection had full symptom remission with ECT, after non-response to trials with multiple medications including olanzapine, risperidone, aripiprazole, sertraline and escitalopram^23^. A second report was that of a 25-year-old who developed a first manic episode with psychosis following COVID-19 infection that was equally resistant to medications but responded well to ECT^24^. Two more COVID-19-related treatment-resistant neuropsychiatric complications both in individuals in their 50s who responded to ECT were reported: a case of malignant catatonia, and the second a case of psychosis and suicidality^25,26^. In some of these cases, there was a notable increase in levels of acute phase reactants, CSF protein and IgG, suggesting a possible immune-mediated mechanism. As the COVID-19 pandemic continues, it is important for clinicians to be aware of the role of ECT as an option for treatment-resistant neuropsychiatric complications associated with COVID-19.

## ECT vs ketamine

Ketamine is emerging as a desirable treatment for treatment-resistant depression (TRD) among many patients since it has fewer cognitive SEs than ECT, and has a rapid onset of action like ECT^27^. Ketamine acts on the glutamate receptor, specifically as an N-methyl-D-aspartate (NMDA) receptor antagonist^27^. Few randomized clinical studies have conducted a head-to-head comparison between ketamine and ECT. Thirty-two patients aged 18 – 59 years old were randomized to receive either twice-weekly IV ketamine infusion or BT-ECT until remission^28^ in a study that reported a more rapid improvement in depression scores in the ketamine treatment arm compared to the ECT treatment arm; however, the effect of ketamine appeared to wear off after about a month of treatment and HDRS score almost returned to baseline by the third month post-treatment. The ECT group, on the other hand, appeared to have a longer-lasting treatment response even at 3 months follow-up and there was no significant difference in cognitive SE between the two treatment groups. The study was limited by a small sample size and it was unclear what severity of depression was used to determine study inclusion and also how long the patients received either treatment. A similar faster antidepressant effect of ketamine compared to ECT was also reported by Basso *et al.* and they additionally found that patients on ketamine had significantly greater improvement in neurocognitive domains of attention and executive function compared to the ECT group, and that verbal memory was significantly lower in the ECT compared to the ketamine group^29^. The study had a very limited follow-up period and hence did not assess whether the antidepressant response was sustainable. 

There may exist an age difference in the treatment effect of ketamine compared to ECT. A much larger study, KetECT, randomized 186 patients with depression to thrice-weekly racemic ketamine or ECT^30^. More patients in the ECT group achieved remission compared to the ketamine group^31^. However, younger patients were noted to have an equally favorable response to ketamine and ECT while older patients responded more favorably to ECT.

Kheirabadi *et al.* explored the role of ECT and ketamine in the treatment of suicidality^32^ by randomizing 45 patients to receive ECT, oral ketamine or intramuscular ketamine, and assessing their depressive and suicidal symptoms using HDRS and BSI at baseline, weekly during the study, and at 1-week and 1-month post-treatment. All three groups had a significant decrease in HDRS and Brief Symptom Inventory (BSI) scores compared to baseline at the end of treatment; however, there was a significant difference in suicidality scores on the BSI in the ketamine treatment groups on day 2 and week 2 of treatment compared to the ECT group. There was no significant difference in the antidepressant effects in the three treatment groups at the end of 1-month follow-up. All the randomized controlled trials (RCTs) discussed above were limited by a small sample size and a short follow-up period. Two larger studies with longer follow-up periods are underway that hope to address these limitations and would provide additional data on the benefits of ketamine versus ECT for maintenance treatment^33,34^. Pooling the available studies, a recent systematic review and meta-analysis found ECT to be more effective than ketamine, and that each intervention had a distinct set of adverse events^35^.

## ECT augmentation

### Reducing side effects

There have been attempts to mitigate the adverse effects of ECT, especially the neurocognitive SEs of ECT. The PRIDE study found a significant decline in cognitive function in several cognitive domains following RUL ultrabrief pulse ECT treatment in geriatric patients with TRD^36^; however, most of these adverse effects resolved within 6 months^16^. Both pharmacological and non-pharmacological interventions have been employed to remediate these adverse effects. A study did not find a clinically significant difference in recall memory and on the mean total scores of the MMSE in patients treated with memantine 5 mg/day vs melatonin 3 mg/day during the first six sessions of ECT treatment^37^. Similarly, donepezil was not found to improve cognitive performance in an RCT that compared donepezil to placebo^38,39^. However, a recent meta-analysis found statistically significant improvement in cognitive function with memantine and liothyronine with moderate to large effect sizes, but the quality of the evidence was generally poor due to insufficient number of studies, small sample size, differences in study methodology and risk of bias^40^. The negative outcomes of these studies may be because a different mechanism, not addressed by these drugs, may underlie the cognitive impairment associated with ECT. An RCT is underway to explore the utility of cognitive control therapy to improve performance by enhancing working memory and cognitive control processes in patients undergoing ECT^41^.

ECT produces a robust hemodynamic response comprising an acute parasympathetic surge, which causes bradycardia followed by a sympathetic surge, which leads to tachycardia and hypertension. Recent studies have explored the role of the α2-adrenergic agonist, dexmedetomidine, in reducing the autonomic response associated with ECT. Two clinical trials have recently reported better hemodynamic response, including lower heart rate, systolic and diastolic blood pressure, mean arterial blood pressure and lower incidence of postictal confusion in patients who were pretreated with dexmedetomidine prior to ECT^42,43^. However, certain drugs that may be co-administered during ECT, such as labetalol and atropine, may hinder the hemodynamic benefits associated with the use of dexmedetomidine^44,45^ and thus caution is warranted in the use of these medications. Despite the potential benefits associated with dexmedetomidine, some authors have noted that it may lower seizure duration, which could affect the quality of ECT^43^.

### ECT plus ketamine augmentation

Studies have reported mixed results regarding the potential benefit of augmenting ECT by adding ketamine as an anesthetic agent by itself or with other anesthetic drugs. A recent meta-analysis pooled data from 17 RCTs that compared ECT + ketamine +/**-** other anesthetic agents versus ECT + other anesthetic agents only^46^ and there was no benefit with ketamine either alone or in combination with other anesthetic drugs. Patients who received ketamine only with ECT were also more likely to have higher blood pressure. The study found no difference between the groups in terms of study discontinuation, recovery time and observed seizure activity. Another meta-analysis that pooled data from 18 RCTs found similar results, with the additional findings of increased EEG seizure duration and reduced ECT dose in patients who received ketamine^47^. Though no difference was found in efficacy and adverse effects in these studies, the potential for ketamine to reduce the dose of ECT may be worth further investigation as a mitigation strategy to reduce overall ECT adverse effects.

### ECT plus transcranial magnetic stimulation (TMS) augmentation

ECT augmentation with interventions such as hyperventilation, use of remifentanil, or even sleep deprivation to lower seizure threshold or increase seizure duration has been explored with the idea that longer seizure duration is associated with superior clinical outcome. TMS applied prior to ECT has been explored as a potential modality of reducing seizure threshold in patients undergoing ECT because of its potential to increase cortical excitability^48^. Buday *et al.* found that this strategy resulted in a significantly lower seizure threshold after administering TMS over the dorsolateral prefrontal cortex (DLPFC) 30–80 minutes prior to ECT treatment^48^. Similarly, Rothermel *et al.* found a significantly greater percentage improvement on the HDRS and that a lower electrical charge was needed for stimulation using this method compared to standard ECT^49^. There was no significant difference in cognitive SEs between treatment groups. The ideal TMS parameters and interval between TMS and ECT treatment needed to produce an optimal decrease in seizure threshold remains unclear.

## Optimizing dosing and administration of ECT

The strength of the electric field induced by conventional ECT is above the excitation threshold of most neuronal tissue in the brain, resulting in activation of almost the entire brain, which may contribute to cognitive SEs^50^. The total charge, pulse width, frequency, train duration and current amplitude all contribute to treatment response, electric field intensity and seizure induction with ECT^51,52^. One area of research has focused on modification of stimulus pulse width to enhance efficacy and reduce cognitive SEs of ECT. Ultrabrief pulse (<0.3 ms) has become popular in clinical practice today due to the evidence for its superior cognitive SEs in RUL ECT. Less is known, however, about its potential benefit with other electrode placements. Two randomized clinical trials have explored its benefit for bitemporal ECT. Results suggest that ultrabrief pulse ECT provides a better cognitive SE profile than brief pulse (1–2 ms) ECT^53,54^. Studies have also indicated a longer treatment course may be needed to achieve remission with ultrabrief pulse ECT^55^.

The amplitude of the pulse has also been an area of research interest. While older studies from the 1940s and 1950s reported on using low current amplitudes to induce seizures, modern studies had left the current amplitude parameter of ECT dosing relatively unexplored until Rosa *et al.* (2011) reported on a case series of five patients successfully treated with 500 mA current^56^. Subsequently, a small pilot study randomized seven patients with depression and active suicidality to low amplitude ECT (500 mA, N=3) versus standard RUL ECT (900 mA, N=4) and all patients achieved remission in suicidal ideation regardless of their treatment group^57^. However, the study was limited by small sample size and absence of data regarding cognitive SEs. Abbott *et al.* (2021) published a larger, well-designed, randomized controlled trial of 60 patients who were randomly assigned to receive ECT at 600, 700, or 800 mA^58^. They found that currents lower than 800 mA had less of an adverse impact on verbal fluency, but the higher currents were associated with better depression outcomes^58^.

The results from these studies indicate that lowering the amplitude of seizure therapy may be a viable option with a suggestion of superior cognitive outcomes, but more studies are needed to confirm these preliminary findings and to identify how to individualize dosing to optimize outcomes. A novel approach to individualizing amplitude is individualized low-amplitude seizure therapy (iLAST), an experimental dosing strategy that aims to deliver an individually titrated current amplitude as a means of achieving therapeutic seizures with a minimal amount of electrical current induced in the brain^59,60^. iLAST is based on the hypothesis that the seizure drives efficacy while the electrical current drives cognitive SEs.

Apart from pulse width and amplitude, spatial position and orientation of the electrodes are equally important contributors to efficacy and adverse effects. BT, RUL and bifrontal (BF) are the three conventional electrode placements commonly used clinically, with the latter two having superior SE profile compared to BT placement. Electrode placements over the temporo-parietal, frontoparietal and supraorbitoparietal regions may have therapeutic advantage over the RUL electrode placement, which remains to be confirmed in more rigorous experimental studies^61^. A randomized cross-over clinical trial that assigned nine participants with TRD to four treatment groups of brief vs ultrabrief and RUL temporoparietal (TP) vs RUL frontoparietal (FP) electrode placements found no superiority of FP over TP placement, but patients who received ultrabrief pulse were found to have better visual memory compared to those who received brief pulse ECT^62^.

Further efforts to enhance control over the electrical stimulation site and distribution of electric field in the brain, such as Focal Electrically-Administered Seizure Therapy (FEAST), use specially shaped electrodes that are placed asymmetrically to enhance focal stimulation over the fronto-orbital cortex^63^. Two recent open-label nonrandomized studies, one that assessed cognitive SEs and antidepressant efficacy and a second that looked at the anti-suicidal efficacy of FEAST compared to standard RUL ECT, found similar efficacy and adverse effect profile with slightly better nonsignificant difference in cognitive adverse effect in the FEAST group^64,65^.

Alternatively, magnetic seizure therapy (MST) uses high doses of TMS, given under anesthesia, to induce therapeutic seizures^66^. MST leverages the focality of TMS (which is confined to superficial cortex) to spare regions of the brain driving cognitive SEs, and couples that with the powerful therapeutic efficacy of seizures. Several clinical studies have compared the efficacy and SE profile of ECT and MST with results showing similar efficacy and better cognitive SE profile with MST^67–69^. More recently, a relatively larger effectiveness study was conducted comparing MST to BT-ECT and RUL-ECT^70^. Patients in the MST group had significantly greater improvement in HDRS-21 score compared to those who received RUL-ECT but not BT-ECT, and time to orientation was significantly shorter in those who received MST compared to ECT treatment groups. Cognitive SEs as measured 4-hours post-session with Wisconsin Card Sorting test and Weschler memory scale-revised favored MST over ECT, but there were no data to determine if these effects were sustainable. Another non-inferiority randomized double blind study is currently underway to compare the efficacy and tolerability of MST compared to RUL-ECT in patients with treatment-resistant depression^71^.

Researchers are challenging the long-held notion that a seizure is required for the antidepressant effect of ECT. Evidence from an open label pilot study in 11 TRD patients who received thrice weekly nonconvulsive electrotherapy (NET) below the seizure threshold were reported to have response and remission rates that were comparable to standard ECT^72^. A randomized clinical trial of transcranial electric stimulation therapy (TEST), which is identical to NET, is underway and aims to deliver low-charge, high-amplitude electrical stimulation by decreasing the frequency and train duration using bifrontal electrodes. Li *et al.* employed a similar technique but termed it low-charge electrotherapy (LCE), where they randomized patients with schizophrenia to receive LCE or standard BT ECT and both groups experienced a significant improvement in Positive and Negative Syndrome Scale (PANSS) score^73^. However, there were significantly lower overall SEs reported in the LCE group. On the other hand, response was slower in the LCE group, and the patients required more treatment sessions. The same group conducted a follow-up RCT study (Hybrid-ECT) by providing the benefits of early remission through initial treatment with standard bitemporal ECT up to four sessions followed by LCE sessions^74^. This study also found comparable improvement in PANNS score between the two groups, but fewer overall SEs and an increased number of treatments in the hybrid-ECT group. Findings from the TEST study would further shed light regarding the therapeutic necessity of a seizure with ECT-like stimulation and may establish the associations between seizure, electrical field magnitude, efficacy and cognitive SEs.

## Conclusion

ECT remains one of the most effective and relatively safe treatments in psychiatry. It is a safe treatment in the elderly population with severe and moderately severe depression, as well as pregnant individuals. However, while there have been positive developments in reducing cognitive SEs with ECT, these SEs still pose a significant barrier to broader utilization. Promising approaches to improve the risk/benefit ratio of ECT include optimization of stimulation parameters and electrode configuration. Investigational interventions such as MST and TEST will help advance our understanding of whether it is the seizure or the electric field that is driving the antidepressant effects of ECT. Further research will be needed to identify who is most likely to benefit from ECT, so that they might receive it earlier in the course of their illness. Multimodal approaches that incorporate tools such as neuroimaging, electrophysiology, electric field modeling and machine learning may enhance our understanding about the mechanism of action of ECT and identify who should receive ECT, as well as identify optimal dosing parameters and electrode placement. We are also in need of research that would explore novel approaches to using ECT that could target a wide range of psychiatric conditions, such as substance use disorders, that are yet to be explored. International collaborations such as International Consortium on the Genetics of Electroconvulsive Therapy and Severe Depressive Disorders (Gen-ECT-ic)^75^ and The Global ECT-MRI Research Collaboration (GEMRIC)^76^ may provide the data needed for further research in these areas. 
